# Language and reading comprehension in middle childhood predicts emotional and behaviour difficulties in adolescence for those with permanent childhood hearing loss

**DOI:** 10.1111/jcpp.12803

**Published:** 2017-09-05

**Authors:** Jim Stevenson, Hannah Pimperton, Jana Kreppner, Sarah Worsfold, Emmanouela Terlektsi, Merle Mahon, Colin Kennedy

**Affiliations:** ^1^ Faculty of Social and Human Sciences University of Southampton Southampton UK; ^2^ Faculty of Medicine University of Southampton Southampton UK; ^3^ School of Education University of Birmingham Birmingham UK; ^4^ Language and Cognition Research Department University College London London UK

**Keywords:** Permanent childhood hearing loss, deaf, reading comprehension, language comprehension, emotional and behaviour difficulties

## Abstract

**Background:**

Permanent childhood hearing loss (PCHL) is associated with an elevated level of emotional and behaviour difficulties (EBD). In children and adolescents with PCHL, EBD has been found to be linked to language ability in children with PCHL. The present study was designed to test whether childhood language and/or reading comprehension abilities of children with PCHL predict subsequent EBD in adolescence.

**Methods:**

Language comprehension (LC) and reading comprehension (RC) were measured at ages 6–10 years (Time 1) and 13–20 years (Time 2) in participants with PCHL who preferred to communicate using spoken language (*n* = 57) and a hearing comparison group (*n* = 38). EBD was measured at both time points by parent and by teacher ratings on the Strengths and Difficulties Questionnaire.

**Results:**

Within the PCHL group there were negative correlations between EBD scores and concurrent LC and RC scores at Time 1 and at Time 2. Cross‐lagged latent variable models fitted to the longitudinal data indicated that the associations between LC, RC and teacher‐rated EBD were more likely to arise from the impact of LC and RC on behaviour rather than the other way around.

**Conclusions:**

In those with PCHL, poor language and reading comprehension in middle childhood increased the risk of emotional and behaviour difficulties at school in the teenage years. The results suggest that effective language and literacy interventions for children with hearing loss may also bring benefits to their mental health.

## Introduction

Children with permanent childhood hearing loss (PCHL) are at risk of developing emotional and behaviour difficulties (EBD) (Stevenson, Kreppner, Pimperton, Worsfold, & Kennedy, 2015; Theunissen et al., 2014). As a group, these children also experience difficulties with expressive and receptive language development (Pimperton & Kennedy, 2012) and the acquisition of reading (Lederberg, Schick, & Spencer, 2013; Moeller, Tomblin, Yoshinaga‐Itano, Connor, & Jerger, 2007), particularly reading comprehension (McCann et al., 2009; Wauters, Van Bon, & Tellings, 2006). However, it is important to recognise that, although at increased risk at a group level, the majority of those with PCHL do not show clinically significant EBD (van Gent, Goedhart, Hindley, & Treffers, 2007; Stevenson et al., in press). In cross‐sectional analyses, we have shown that in middle childhood (Stevenson, McCann, Watkin, Worsfold, & Kennedy, 2010) and in adolescence (Stevenson et al., 2017) that within the PCHL group those with less well developed receptive language are more likely to have EBD.

The focus of this paper will be to use longitudinal data obtained in childhood and in adolescence to examine the relationship over time between EBD and language and reading comprehension in a cohort of young people with PCHL.

### Language and behaviour

In the preschool child differences, there is evidence of reciprocal associations between conduct problems and expressive language between those 3 and 5 years of age (Girard, Pingault, Doyle, Falissard, & Tremblay, 2016).

There is substantial stability in individual differences in language ability up to age 11 years Bornstein, Hahn, and Putnick (2016). In part, this explains why behavioural difference such as those reflected in temperament variation have not been found to relate to individual differences in receptive vocabulary growth between 4 and 8 years of age (Taylor, Chritensen, Lawrence, Mitrou, & Zubrock, 2013). In the general population there is a relative dearth of studies that explicitly examine the direction of effects in the association between behaviour problems and language ability in children after the preschool period (Conti‐Ramsden, 2013). However, a pair of such studies was reported by Petersen et al. (2013). They modelled the cross‐lagged relationships between behaviour problems and language ability in a sample of 585 children followed between the ages of 7 and 13 years. The second sample contained 11,506 children who were followed were between 4 and 12 years of age. In both samples after controlling for autoregressive effects they found that the effect of language ability on later behaviour problems was much stronger than that of behaviour on language.

A number of studies have addressed the question of the relationship between language and EBD by studying children with children with developmental language disorder (DLD; formerly called specific language impairment, Bishop, Snowling, Thompson, & Greenhalgh, 2016)). A systematic review and meta‐analysis by Yew and O'Kearney (2013) found that children with DLD were approximately twice as likely as typically developing children to show disordered levels of overall internalising problems, overall externalising and ADHD problems.

St Clair, Pickles, Durkin, and Conti‐Ramsden (2011) investigated the relationship between both reading and language skills and EBD in a cohort of 234 children with DLD. Of these, 103 were reassessed at later ages through to age 16 years. EBD was assessed with the Strengths and Difficulties Questionnaire (SDQ) (Goodman, 1997). They found that reading skills and expressive language measured at age 7 years were related to later behaviour difficulties. Early language comprehension measured using the Test for Reception of Grammar (TROG) (Bishop, 2003) was not related to later SDQ scores, but pragmatic language skills did show such a relationship.

Studies of children and adolescents with psychiatric disorders have also aimed to examine the relationship between EBD and language. For example, Cohen, Farnia, and Im‐Bolter (2013) showed that a sample of 144 adolescents who had been referred to mental health services had language scores significantly below those of a comparison sample of 186 who had not had such a referral.

The relationship between language and EBD has also been studied in children who are deaf (see review by Gentili & Holwell, 2011)). Studies of children with PCHL assessed below the age of 2 years found that they showed more EBD than hearing children (e.g. Topol, Girard, St Pierre, Tucker, & Vohr, 2011). At this young age, language skill deficits in the PCHL children were not related to high EBD score. However, after the age of 3 years, language abilities in deaf children have been found to be closely related to their social functioning and behaviour (Barker, Quittner, Fink, Tobey, & Niparko, 2009; Meinzen‐Derr et al., 2014). The same association between language ability and behaviour difficulty, specifically peer relationship problems, has also been found in deaf adolescents (Fellinger, Holzinger, Beitel, Laucht, & Goldberg, 2009).

### Reading ability and behaviour

In reviewing early studies on the relationships between externalising EBD and reading problems, Hinshaw (1992) concluded ‘the overlap between externalising difficulties and academic failure clearly is sizable and important.’

More recently a large‐scale cross‐sectional UK survey was used to examine the association between literacy difficulties and EBD (Carroll, Maughan, Goodman, & Meltzer, 2005). The results suggested that externalising problems were associated with literacy difficulties and this effect was mediated via inattentiveness. In contrast, the pattern of results suggested a direct association between literacy difficulties and anxiety.

Fergusson and Lynskey (1997) showed the importance of controlling for prior behaviour when examining relationships between reading and EBD. They found that although early reading problems at age 8 years were associated with later conduct problems, this association no longer held when appropriate controls for confounding variables were introduced. In particular, they emphasised the importance of controlling for early behaviour problems. However, Bennett, Brown, Boyle, Racine, and Offord (2003) reached a different conclusion. They conducted a similar study that took into account a wide range of potentially confounding factors, including the initial level of conduct problems. They suggest that low reading achievement at school entry may contribute to later conduct problems. Trzesniewski, Moffitt, Caspi, Taylor, and Maughan (2006) used a cross‐lagged analysis in a longitudinally studied sample of twins representative of the UK population. They found that there was evidenced in both boys and girls that antisocial behaviour at 5 years was influencing reading at age 7 years when autoregressive effects were controlled.

There have been few studies that have investigated the relationship between EBD and reading ability in deaf children. In a cross‐sectional analysis, Calderon (2000) found that in 28 children with prelingual hearing loss of >55 dB HL aged between 45 and 88 months, the correlation between reading ability and externalising behaviour was *r* = −.48 (*p* < .01).

### Summary of previous studies

Therefore, for the general population, for those with DLD and for deaf children there is replicated evidence suggesting a strong concurrent association between language and reading ability and EBD. However, cross‐sectional studies cannot provide insights into the direction of the language‐EBD relationship (i.e. whether earlier language or reading deficits lead to later emotional and behavioural problems, or vice‐versa). There is a need for further longitudinal studies of this association in deaf children and particularly for studies that extend the age range into adolescence.

### The present study

In this paper, we present a longitudinal analysis of the relationships between language and reading comprehension and EBD between the ages of 6–10 and 13–20 years in children with PCHL. We have previously shown in this sample that those with PCHL had mean reading levels significantly below those of the hearing comparison group (HCG) at both age groups 6–10 years (McCann et al., 2009) and 13–20 years (Pimperton et al., 2016). Similar findings have also been reported for language comprehension scores (Kennedy et al., 2006; Pimperton et al., 2017). Reading deficits for teenagers with PCHL were most severe in the domain of comprehension (Pimperton et al., 2016). Those with PCHL also showed significantly higher scores on the SDQ than the HCG at both age groups 6–10 years (Stevenson et al., 2011) and 13–19 years (Stevenson et al., 2017). In addition, at both these age groups there was a strong association between the behaviour and receptive language abilities of the PCHL participants (Stevenson et al., 2010; Stevenson et al., 2017). Indeed, the elevated rate of behaviour problems shown by those with PCHL compared to the HCG was reduced to nonsignificance once differences in receptive language ability were taken into account. For these reasons the analyses to be presented here will be based on comprehension measures of both language and reading.

The analysis being undertaken allows adjustment for behaviour at ages 6–10 years and therefore can provide a more direct test of the effects of early language on behaviour at 13–20 years. This design feature has been argued to be of value in the analysis of the longitudinal relationship between language and behaviour in children with specific language impairment (Yew & O'Kearney, 2013).

Based on previous studies reviewed above, we anticipated that we would find that both language comprehension and reading comprehension are significantly associated with EBD in adolescence. Furthermore, based on the findings of Bennett et al. (2003) and St Clair et al. (2011), we expected that a longitudinal analysis would show that the language and reading measures would be predictive of later EBD. We expected that EBD would not be a predictor of later language (Petersen et al., 2013) but that it may predict later reading (Trzesniewski et al., 2006).

## Methods

### Participants

The participants who provided data for this study are part of a population‐based cohort study of children with PCHL (Kennedy et al., 2006). Participants in the cohort study were drawn from a 1992–1997 birth cohort of 157,000 children born in eight districts of southern England and 120 participated in the previous phase of the study aged 6–10 years (Kennedy et al., 2006; McCann et al., 2009; Stevenson et al., 2011). All had been diagnosed by age 7 with a PCHL ≥40 dB in the better ear that was not known to be of postnatal onset. A HCG (*n* = 63) that was half the size of the group of participants with PCHL was also included in the previous phase of the study. To derive this HCG for every two participants with PCHL, a child with the same place and closely similar date of birth was randomly selected and the family were approached for their consent to their child's inclusion in the HCG for this study. In the current phase of the study (aged 13–19 years), 76 of the 120 eligible participants from the PCHL group (63%) and 38 of the 63 eligible participants from the HCG (60%) were contactable and agreed to participate.

### Attrition

The annual attrition rate among children with PCHL eligible was 3% over 17 years since their initial recruitment and 4% over the 9 years since their assessment at primary school. This degree of attrition is relatively low for follow‐up studies of long‐term paediatric conditions (Karlson & Rapoff, 2009). Attrition was largely attributable to the participants not responding to requests to participate in the later phase of the study.

All 38 HCG teenagers completed both the reading comprehension and language comprehension assessments. Of the 76 teenagers with PCHL, 66 and 63 completed the reading comprehension and receptive language assessments respectively. Those who did not complete the assessments either had severe additional disabilities that precluded the development of sufficient language to attempt the test, or in the case of the receptive language comprehension assessments, used British Sign Language as their preferred language, hence these spoken English assessments were not appropriate for them. The results of the study are therefore only generalisable to those with PCHL who prefer to communicate using spoken language. There were 57 children in the PCHL group who had both language and reading comprehension measures at both Time 1 and Time 2. The analyses in this paper were restricted to these 57 children so that the analyses could be directly comparable for the language and reading measures since they were based on the same group of participants.

The mean Time 1 parent rating SDQ Total Difficulties in the PCHI group (*n* = 57) was lower than that for those spoken language users who did not take part at Time 2 [(*n* = 43); mean difference = −2.62, 95% CI −5.12 to −0.04]. There was no significant difference in the Time 1 teacher Total Difficulties means for these groups (mean difference = −1.39, 95% CI −3.94 to 1.17). Similarly there were no significant differences between these groups in either their Time 1 Language Comprehension (mean difference = 0.06, 95% CI −0.54 to 0.67) or Reading Comprehension scores (mean difference = 0.10, 95% CI −0.41 to 0.61).

### Procedure

Participants were assessed by a trained researcher who was unaware of their audiological history (e.g. the age at which the hearing loss of the children with PCHL was confirmed). A number of identifying factors meant that it was not feasible to blind the researchers to whether the child had PCHL. The assessment session was undertaken in a quiet room at their home or school. The following measures were administered:

#### Language comprehension (LC)

The Test for Reception of Grammar Version 2 (TROG‐2; Bishop, 2003) was used to assess participants’ receptive skills for spoken English grammar, and the British Picture Vocabulary Scale Third Edition (BPVS‐3; Dunn, Dunn & National Foundation for Educational Research, 2009) provided a measure of their receptive skills for spoken English vocabulary. Both of these assessments were used to measure the participants’ LC skills at Time 1 and at Time 2, although earlier versions of the assessments were used at Time 1.

#### Reading comprehension (RC)

Reading comprehension was assessed using standardised tests of reading comprehension at both time points. At Time 1, the Wechsler Objective Reading Dimensions (WORD) (Wechsler, 2005) was used. At Time 2, the York Assessment of Reading for Comprehension Secondary Edition (YARC), a standardised reading test for secondary school‐aged children (Stothard, Hulme, Clarke, Barmby, & Snowling, 2010) was administered.

#### Nonverbal ability (N‐VA)

Nonverbal ability was assessed at Time 1 using the Raven's Standard Progressive Matrices (Styles, Raven, & Raven, 1998).

#### Behaviour

Emotional and behaviour difficulties were measured with teacher and parent versions of the SDQ (Goodman, 1997). This is a widely used behaviour screening questionnaire on children and young people's behaviours, emotions and relationships. It has been recommended as suitable for use with children with PCHL (Hintermair, 2007). A Total Difficulties score reflecting EBD was derived from summing the scores of four SDQ scales (Emotional Symptoms, Conduct Problems, Hyperactivity and Peer Problems) on the parent and teacher questionnaires separately.

#### Other characteristics

Other characteristics of the teenager and their family, including maternal education level and languages used in the home, were also documented. Audiological data were collected from audiology and cochlear implant centres. Severity of hearing loss was categorised from the most recent audiological records as moderate (40–69 dB HL), severe (70–94 dB HL) or profound (≥95 dB HL) according to four‐frequency averaging of the pure‐tone thresholds from 500 to 4000 Hz.

### Analysis strategy

For comparisons within the group of teenagers with PCHL, we used norms obtained from the HCG (Kennedy et al., 2006). The group mean score and standard deviation scores for the HCG on each measure were used to derive age‐adjusted z scores for the teenagers with PCHL on that measure. A composite LC measure was generated using these z scores by averaging the BPVS and TROG z scores. These two measures were highly correlated (*r* = .79, 95% CI 0.65 to 0.88). EBD was analysed using SDQ raw scores.

To examine the longitudinal relationships between language and reading comprehension and behaviour, structural equation modelling was conducted in STATA 13 (StataCorp, 2013). This analysis was limited to the PCHL group as the sample size for the HCG was too small to provide adequate power to test these models. Maximum likelihood methods were used for parameter estimation and for imputing sporadic missing data (Graham, 2009). The distributions of the parent‐ and teacher‐rated SDQ scores were positively skewed with a longer tail in the high scores, as is usually found with this questionnaire (Hill et al., 2016). To address this issue, bootstrapped estimates of the standard errors were obtained (Russell & Dean, 2000). We also calculated robust estimates of the standard errors but only report the 95% CI for the bootstrap estimates as the two methods produced very similar estimates.

The longitudinal analysis of the cross‐lagged relationships used latent variables as recommended by Cole and Preacher (2014). Using the ‘reliability’ option in STATA, latent variables were created for the comprehension and EBD measures at Time 1 and Time 2. The error variance for each indicator is based on the reliability of the manifest variables. By this means, only true score variances are used to estimate regressions and correlations between the constructs.

Reliabilities were not available for deaf children on these measures. However, where studies have been conducted for the deaf, the reliabilities have been found to be as good, if not higher, than those of typically developing children: on a test of written grammar (Cannon, Hubley, Millhoff, & Mazlouman, 2016), on WISC IQ (Krous & Braden, 2011) and on Generalizable Skills Assessments (Loeding & Greenan, 1998). The reliabilities for the tests used were therefore taken from studies on typically developing children as follows: BPVS α = .93, (Dunn, Dunn, Whetton, & Burley,^1997^); TROG‐2 split‐half = 0.88 (Bishop, 2003); WORD comprehension split‐half = 0.91 (Wechsler, 2005); YARC mean α = .87 (Stothard et al., 2010); parent‐rated SDQ Total Score α = .82 (Goodman, 2001); teacher‐rated SDQ Total Score α = .87 (Goodman, 2001); Raven's Progressive Matrices split‐half = 0.94 (Styles et al., 1998).

Accordingly, the following values were used as the reliability estimates: Time 1 and Time 2 parent SDQ (0.82), Time 1 and Time 2 teacher SDQ (0.87), Time 1 Reading comprehension (0.91), Time 2 Reading comprehension (0.87), Time 1 and Time 2 Language comprehension (0.90) (average of BPVS and TROG‐2), Time 1 Raven's Progressive Matrices (RPM) (0.94).

The standardised path coefficients and associated 95% CI are presented for the saturated model and for a trimmed final model, which retains just those coefficients whose 95% CI do not include zero. This model trimming has the advantage of providing more precise parameter estimates (Bentler & Mooijaart, 1989).

### Ethical approval

This study was approved by the Southampton and South West Hampshire Research Ethics Committee. Written informed consent for participation in the study was obtained from principal caregivers and from the teenage participants.

## Results

### Demographic characteristics

The demographic characteristics of the PCHI (*n* = 57) and HCG (*n* = 38) participants are presented in Table 1. There were no significant differences between the PCHL and HCG on mean age at Time 1, gender, English as the main language at home, maternal education and occupation of head of household (see Table 1). At Time 2 the PCHL group was significantly older than the HCG (mean difference = 0.83 95% CI 0.27 to 1.38). However, the correlation between age and the Time 2 SDQ Total scores was not significant within either the PCHL group or the HCG and so no between‐groups adjustments were made for age. The language and reading comprehension scores were age adjusted.

**Table 1:** Demographic characteristics of participating children with PCHL and of a Hearing Comparison Group

Characteristic	Participants with PCHL *n* = 57	HCG *n* = 38	PCHL versus HCG
Mean age (*SD*) [range] at Time 1	7.93 (1.05) [6.42 to 10.67]	8.02 (1.08) [6.25 to 9.75]	*t* = 0.38, *df* = 93, *p* = .703
Mean age (*SD*) [range] at Time 2	17.17 (1.41) [14.67 to 20.42]	16.34 (1.24) [14.17 to 19.00]	*t* = 2.93, *df* = 93, *p* = .004
Female sex *n* (%)	28 (49.1)	13 (34.2)	χ^2^ = 2.07, *df* = 1, *p* = .151
Severity of hearing loss *n* (%)			
Moderate	30 (52.6)	NA	NA
Severe	13 (22.8)	NA	
Profound	14 (24.6)	NA	
English as main language at home *n* (%)	51 (89.5)	36 (94.7)	χ^2^ = 0.82, *df* = 1, *p* = .365
Maternal education *n* (%)			
No qualifications or <5 O‐levels^a^	16 (28.1)	11 (28.9)	χ^2^ = 3.64, *df* = 2, *p* = .162
5 O‐levels or some A‐levels^a^	33 (57.9)	16 (42.1)	
University or higher degree	8 (14.0)	11 (28.9)	
Occupation of head of household^b^ *n* (%)			
Never worked/unemployed	9 (15.8)	1 (2.6)	χ^2^ = 5.10, *df* = 3, *p* = .164
Lower occupations	5 (8.8)	3 (7.9)	
Intermediate occupations	14 (24.6)	8 (21.1)	
Higher occupations	29 (50.69)	26 (68.4)	

PCHL = Permanent childhood hearing loss ≥40 dB in the better ear; HCG = Hearing comparison group; NA = Not applicable.

a O‐level examinations (now replaced by General Certificates of Education) are usually taken at 16 years of age: A‐level examinations (now replaced by A2s) are taken 2 years later as qualifications for entry to higher education.

b Classified as per 2001 UK census.

### Mean scores at Time 1 and Time 2 for the PCHL sample

The means on the language, reading and EBD variables for those for whom measures at both Time 1 and Time 2 were available are given in Table 2. The mean z scores on both LC and RC showed little change with age and this was not significant. There were decreases in the Total Difficulties score rated by parents and for teachers this was significant (Standardised mean difference (SMD) = 0.51, 95% CI 0.07 to 0.95).

**Table 2:** Differences between Time 1 (aged 6–10 years) and Time 2 (aged 13–20 years) group mean language comprehension and reading comprehension *z* scores and EBD scores for children with PCHL

	*n*	Time 1 (T1)		Time 2 (T2)		T2–T1	Paired *t*‐test for difference in means (T2–T1)		
		Mean	*SD*	Mean	*SD*	SMD (95% CI)	*t*	*df*	*p*
Language comprehension	57	−2.07	1.55	−1.88	2.41	−0.09 (−0.46 to 0.27)	0.96	56	.50
Reading comprehension	57	−0.91	1.17	−1.09	1.49	0.13 (−0.23 to 0.50)	1.24	56	.10
Parent‐rated SDQ total	55	7.58	4.78	7.00	5.15	0.11 (−0.26 to 0.49)	0.75	54	.18
Teacher‐rated SDQ total	41	7.80	5.89	5.12	4.42	0.51 (0.07 to 0.95)	3.22	40	.01

EBD = Emotional and behavioural disorders; PCHL = Permanent childhood hearing loss ≥40 dB in better ear; *SD* = Standard Deviation; SMD = Standardised mean difference; T1 = Time 1; T2 = Time 2; SDQ = Strengths and Difficulties Questionnaire.

### Associations between language and reading comprehension and EBD scores in the PCHL sample

The correlations between language and reading comprehension and the SDQ Total Difficulties scores at Time 1 and Time 2 are presented in Table 3.

**Table 3:** Correlations between language comprehension, reading comprehension, and parent‐rated and teacher‐rated EBD scores at Time 1 (aged 6–10 years) and Time 2 (aged 13–20 years) in children with PCHL (*n* = 57)

	1.	2.	3.	4.	5.	6.
Parent‐rated SDQ Total Difficulties scores						
At Time 1						
1. LC	1.00					
2. RC	.84***	1.00				
3. SDQ total	−.17	−.25*	1.00			
At Time 2						
4. LC	.78**	.67**	−.15	1.00		
5. RC	.75**	.67**	−.10	.74**	1.00	
6. SDQ total	−.24	−.19	.37**	−.21	−.26*	1.00
Teacher‐rated SDQ Total Difficulties scores						
At Time 1						
1. LC	1.00					
2. RC	.84**	1.00				
3. SDQ total	−.22	−.38**	1.00			
At Time 2						
4. LC	.78**	.67**	−.17	1.00		
5. RC	.75***	.67**	−.24	.74**	1.00	
6. SDQ total	−.32*	−.47*	.49**	−.29	−.20	1.00

EBD = Emotional and behavioural disorders; PCHL = Permanent childhood hearing loss ≥40 dB in the better ear; SDQ = Strengths and Difficulties Questionnaire; LC = Language Comprehension; RC = Reading Comprehension.

*p < .05; **p < .01; ***p < .001.

At both Time 1 and Time 2 there were significant correlations between concurrent scores for LC and RC. For both parent and teacher ratings the Total Difficulties EBD score was negatively associated with concurrent LC and RC scores at both time points, but these correlations were not significant in every case. The correlation between LC and RC at Time 1 and Time 2 teacher‐rated EBD at Time 2 were significant.

### Cross‐lagged models of the relationship between Time 1 and Time 2 scores for the PCHL sample

**Figure 1:**
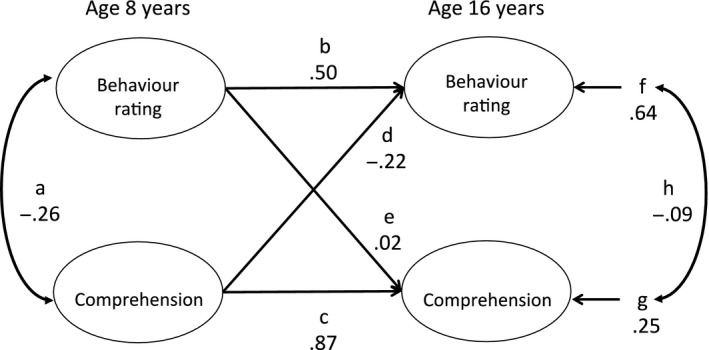
Schematic diagram of the latent variable models of behaviour and comprehension at age 8 and 16 years. Path labels correspond to entries in Table 4. Path coefficients are illustrated with values from the saturated model of teacher ratings of emotional and behaviour difficulties (EBD) and language comprehension

To examine the longitudinal relations between the LC, RC and the EBD measures at Time 1 and at Time 2 (*n* = 57) cross‐lagged models were tested. The same participants were included in both models and in each case separate models were tested for parent‐ and teacher‐rated SDQ scores. These models incorporate the concurrent association at Time 1, cross‐lagged relationships and the degree of stability between Time 1 and Time 2 for LC and SDQ Total score and RC and SDQ Total score separately for parent and teacher ratings. The correlated error terms for the Time 2‐dependent latent variables represent the extent to which unexplained variance (error) in these measures is correlated. These models are presented schematically in Figure 1. The results will be discussed in relation to the Final (trimmed) model in each case with paths removed that included zero in their 95% CIs.

As a check on the effects of missing data for the Time 2 teacher‐rated EBD measure the means on the RC and LC measures at Time 1 and Time 2 were compared for those with and without Time 2 teacher‐rated SDQ score. A MANOVA for these four measures showed no significant differences between these groups (Wilk's Lamda = 0.86, Multivariate *F* = 2.15, *df* = 4, 52, *p* = .09).

For the LC measure the pattern of results was similar for parent and teacher ratings of behaviour (Table 4). The stability of the behaviour ratings over time (path b) was lower than the stability of the LC measures (path c). The error terms were not significantly correlated (path h) for either the parent or teacher ratings. The key test of the putative direction of causality is the size of the cross‐lagged paths. For both the parent‐ and teacher‐rated behaviour models the paths from Time 1 behaviour to Time 2 LC (path e) were not significant. The paths in the other direction (path d) were larger but for parent‐rated behaviour this was not significantly different from zero (−0.20, 95% CI −0.44 to 0.03). For teacher ratings, a significant coefficient of −.26 (95% CI −0.51 to −0.02) was obtained for path d. Thus, a low score on the LC measure at Time 1 was associated with a high SDQ Total Difficulties score at Time 2, when Time 1 SDQ Total Difficulties scores were taken into account.

**Table 4:** Coefficients in cross‐lagged latent variable models of Time 1 and Time 2 parent and teacher ratings of EBD and comprehension abilities in 57 children and adolescents with PCHL

	Standardised coefficient and 95% CI Paths as labelled in Figure 1							
	A	b	c	d	e	f	g	h
Parent rating and language comprehension								
Saturated model	−0.19	0.39	0.87	−0.20	0.01	0.77	0.25	−0.07
Bootstrap 95% CI	−0.41 to −0.03	0.04 to 0.75	0.75 to 0.98	−0.47 to 0.07	−0.19 to 0.18	0.56 to 1.06	0.11 to 0.54	−1.37 to 1.21
Final model	Fixed 0	0.44	0.86	Fixed 0	Fixed 0	0.80	0.24	Fixed 0
LR test of final model versus Saturated χ^2^ = 5.74, *df* = 4, *p* = .219								
Bootstrap 95% CI	–	0.11 to 0.67	0.75 to 0.99	–	–	0.57 to 1.14	0.11 to 0.56	–
Teacher rating and language comprehension								
Saturated model	−0.26	0.50	0.87	−0.22	0.02	0.64	0.25	−0.09
Bootstrap 95% CI	−0.53 to 0.00	0.09 to 0.89	0.75 to 0.99	−0.51 to −0.05	−0.15 to 0.18	0.35 to 1.09	0.11 to 0.54	−1.43 to 1.25
Final model	Fixed 0	0.49	0.87	−0.26	Fixed 0	0.68	0.24	Fixed 0
LR test of final model versus saturated χ^2^ = 3.11 *df* = 3, *p* = .376								
Bootstrap 95% CI	–	0.11 to 0.88	0.75 to 0.98	−0.51 to −0.02	–	0.40 to 1.14	0.11 to 0.55	–
Parent rating and reading comprehension								
Saturated model	−0.29	0.40	0.78	−0.11	0.11	0.79	0.42	−0.94
Bootstrap 95% CI	−0.52 to −0.07	0.04 to 0.77	0.63 to 0.94	−0.37 to 0.15	−0.13 to 0.35	0.57 to 1.12	0.26 to 0.67	−2.04 to 0.14
Final model	−0.29	0.44	0.75	Fixed 0	Fixed 0	0.80	0.43	Fixed 0
LR test of final model versus saturated χ^2^ = 3.93, *df* = 3, *p* = .270								
Bootstrap 95% CI	−0.51 to −0.07	0.13 to 0.75	0.62 to 0.88	–	–	0.57 to 1.13	0.27 to 0.68	–
Teacher rating and reading comprehension								
Saturated model	−0.44	0.40	0.78	−0.36	0.07	0.58	0.39	0.96
Bootstrap 95% CI	−0.76 to −0.12	−0.04 to 0.85	0.57 to 0.99	−0.69 to −0.03	−0.30 to 0.43	0.31 to 1.07	0.27 to 0.68	−0.24 to 2.17
Final model	−0.44	0.42^a^	0.75	−0.33	Fixed 0	0.58	0.44	Fixed 0
LR test of final model versus saturated χ^2^ = 2.14, *df* = 2, *p* = .342								
Bootstrap 95% CI	−0.75 to −0.12	−0.01 to 0.86	0.61 to 0.88	−0.66 to −0.00	–	0.32 to 1.07	0.28 to 0.69	–

EBD = Emotional and behavioural disorders; PCHL = Permanent childhood hearing loss ≥40 dB in the better ear.

Strengths and Difficulties Questionnaire Total Difficulties scores were used as the EBD measure.

a Dropping this term resulted in a significant increase in χ^2^.

For the RC measures for both the parent‐ and teacher‐rated behaviour models the paths from Time 1 behaviour to Time 2 RC (path e) were not significant. On parent ratings, Time 1 RC was not significantly associated with Total Difficulties score at Time 2. However, the teacher‐rated scores showed a significant cross‐lagged effect from Time 1 RC to Time 2 behaviour (−0.36, 95% CI −0.69 to −0.03).

As a test for the specificity of the relationship between the language and reading comprehension measures at Time 1 and teacher ratings of EBD at Time 2, these models were reanalysed with N‐VA at Time 1 as an additional latent variable predictor of Time 2 teacher ratings. The paths from N‐VA to teacher Time 2 ratings were not significant in both the LC model (β = −.11, 95% CI −0.39 to 0.14) and the RC model (β = −.07, 95% CI −0.37 to 0.22). The paths from LC (β = −.22, 95% CI −0.51 to 0.08) and from RC (β = −.34, 95% CI −0.68 to −0.00) to teacher Time 2 EBD were largely unchanged. These results suggest that verbal rather than more general cognitive abilities are the abilities that are related to school‐based EBD.

## Discussion

Longitudinal relationships were found between language and reading comprehension measures in middle childhood and EBD rated by teachers in adolescence. The cross‐lagged analysis showed that language and reading measured in childhood predicted behaviour during the teenage years even when the continuities in behaviour are taken into account. Nonverbal ability in childhood was not predictive of behaviour in the teenage years. This longitudinal analysis suggests that comprehension abilities may influence the development of EBD in children with PCHL. The size of the standardised regression coefficients between the Time 1 comprehension scores and Time 2 EBD scores are modest. However, it is particularly striking that this relationship should be identified in a prospective study with a 9‐year interval between assessments.

These longitudinal findings should be treated with some caution since cross‐lagged analyses cannot definitively establish causality (Card & Little, 2007) and applying a cross‐lagged model to just two time points provides only a weak test of putative direction of causality (Newsom, 2012). Future studies should aim to replicate these findings using multiple assessment time points within a cohort of participants with PCHL.

It should be noted that the childhood comprehension measures (both reading and language) were only significantly related to behaviour in the teenage years as rated by teachers. The correlations over time for the behaviour measures were similar for parent and teacher ratings and accordingly this finding cannot be explained by differences in stability for the two sets of behaviour ratings. It seems likely that the child's behaviour at school is more closely related to comprehension measures than is behaviour at home. This is indicated by the correlations at Time 1 between the comprehension measures (especially reading) and teacher ratings of EBD being larger than parent behaviour ratings. It is of interest to note that in the large general population sample studied by Carroll et al. (2005), the scores on the SDQ for those with specific literacy difficulties were significantly higher than those of controls on both teacher and parent ratings. It may therefore be that language and reading comprehension are more specifically associated with school‐based behaviour problems in the deaf compared to typically developing children.

There are a number of possible mechanisms that could produce a link between comprehension abilities and EBD in those with PCHL (see Gentili & Holwell, 2011 for an overview). These include the possible impact of language comprehension difficulties on mediators such as theory of mind, executive function and emotional regulation. Language comprehension also may be associated with a lack of facility in the use of inner speech. Inner speech has been postulated as an important component to literacy development that may be compromised in the deaf population (Mayer & Wells, 1996). There is only limited research on the inner speech of deaf individuals and no studies have related individual differences in the use of inner speech to the presence of EBD in this population (Alderson‐Day & Fernyhough, 2015). There is some evidence that for young hearing children individual differences in the development of inner speech is related to externalising behaviour problems (Winsler, De León, Wallace, Carlton, & Willson‐Quayle, 2003). In the present study, no measure of inner speech was taken and the possible significance of inner speech for the development of self‐regulation in the deaf requires further examination.

The results reported here are broadly consistent with those of St Clair et al. (2011) in their study using participants with DLD in showing a longitudinal relationship between language and EBD but the present findings indicate that different sources of language impairments (PCHL vs. DLD) may be associated with some differences in language‐behaviour relationships. For those with PCHL, receptive aspects of language ability may have a more marked role in leading to EBD than for children with language impairments. In those with DLD, other aspects of language may be more salient, for example, pragmatic and expressive language ability (St Clair et al., 2011).

Those with PCHL will clearly have additional influences on their LC and RC abilities that are not specific to children with hearing loss. As in other children, these comprehension abilities will be subject to effects stemming from genetic differences (Dale et al., 1998; Gialluisi et al., 2014) and social experiences (Beitchman et al., 2008). Nevertheless, the present findings indicate that effective interventions focused on enhancing the receptive language abilities of children with PCHL may have subsequent benefits in terms of reducing EBD.

This conclusion supports the argument made by Gentili and Holwell (2011) concerning the crucial need for efficient language acquisition in those with PCHL to reduce the risk of mental health problems. There are intervention programmes available for those with speech, language and communication needs, although the evidence base for their effectiveness is weak (Dockrell, Lindsay, Roulstone, & Law, 2014). The evidence on interventions to support the language development of deaf children suggests that early intervention with active family involvement is likely to be most effective (Moeller, 2000) and a variety of such approaches have been developed (Rees et al., 2015). The beneficial impact of early intervention on language for children with permanent hearing loss has been demonstrated (Meinzen‐Derr, Wiley, & Choo, 2011). However, there is a paucity of evaluation studies to identify optimal intervention strategies to enhance language in the deaf population (Lederberg et al., 2013).

The findings of the present study also suggest that interventions designed to improve the reading ability of deaf children may additionally be effective in reducing the risk of EBD. As with language intervention, there is no good quality evidence for the efficacy of specific types of reading instruction for deaf children (i.e. which approaches are effective in supporting deaf children's reading development). Luckner, Sehakl, Ctxiney, Young, and Muir (2005/2006) attempted to produce a meta‐analysis of studies on the effectiveness of reading instruction for deaf children but they concluded that the evidence base was not sufficiently strong for a quantitative analysis of effect sizes to be undertaken. A more recent review of quantitative and qualitative meta‐analyses on reading research concluded that there is still a paucity of high‐quality research on deaf children and that there is not yet an adequate basis to determine evidence‐based practices for reading instruction for the deaf, although some instruction approaches are suggested to warrant further investigation (Wang & Williams, 2014). There is clearly a need for high‐quality, methodologically rigorous studies to assess the value of specific interventions in supporting deaf children's reading development in order to build the evidence base in this area.

The present study had a number of limitations. The findings in this paper are based only on those with PCHL who prefer to communicate using spoken language. It would have been preferable to have comprehension and EBD measures available on more than two occasions to adequately test for the direction of effects, as discussed above. Additionally, the sample size for the HCG was too small to allow a test for differences in the path models for the PCHL group and the HCG, meaning that comparisons between the two groups within this cohort could not be made. The small sample size for the PCHI group (*n* = 57) potentially presents a problem of low power. However, the strategy of employing models that use single indicator latent measures with reliabilities ranging from 0.82 to 0.94 means that power is substantially improved over an analysis based on observed variables (Wolf, Harrington, Clark, & Miller, 2013). Finally, there were no measures of possibly important social influences (e.g. parental involvement) on both comprehension and EBD available in the present study. It has been shown, for example, that parent‐based interventions that is, shared book reading, conversations and writing interactions – are effective in enhancing the language and early literacy skills of preschool children. (Reese, Sparks, & Leyva, 2010). Future research should address the three‐way relationships between the quality of parent/child interaction, language development and EBD in deaf children.

With due considerations to these limitations, the present study provides evidence that comprehension measures (but not nonverbal ability) in middle childhood are predictive of teacher‐rated EBD in the teenage years, whereas EBD in childhood does not predict later comprehension. The findings reported in this paper add weight to this need to identify effective language and literacy interventions for deaf children, not least because of the possible benefits to their mental health.

**Key points**

- Those with permanent childhood hearing loss (PCHL) are likely to show more emotional and behaviour difficulties (EBD) than their hearing peers.
- PCHL is associated with deficits in language comprehension and reading comprehension.
- In those with PCHL, low language and reading scores in middle childhood are predictive of teacher‐rated EBD in their teenage years. However, a causal relationship has yet to be definitively established.
- Intervention to support the language and literacy of the deaf and hard of hearing may additionally benefit their mental health.
